# Molecular biology of the gut

**DOI:** 10.1186/s40348-016-0059-1

**Published:** 2016-08-12

**Authors:** Hassan Y. Naim, Klaus-Peter Zimmer, Buford Nichols

**Affiliations:** 1 Department of Physiological Chemistry, University of Veterinary Medicine Hannover, Bünteweg17, D-30559 Hannover, Germany; 2 Department of General Pediatrics and Neonatology, Center for Pediatrics and Adolescent Medicine, Justus-Liebig-University Giessen, Feulgenstr. 12, D-35392 Giessen, Germany; 3 Baylor College of Medicine, 1 Baylor Plz, Houston, TX 77030 USA

## Editorial

The gastrointestinal tract is the largest exposed surface among mammalian macroorganisms. It is constantly exposed to substantial challenges and exerts a potpourri of diverse physiological functions. Among the major intestinal functions is the digestion of nutrients and subsequent uptake and metabolism of their respective building blocks, control of absorption and secretion of water, salts and nutrients, interaction of the mucosal surface with micro-organisms and cross-talk with the submucosa, and communication at the basolateral surface with cells of innate and adaptive immune system. These functions are tightly linked to a large network of protein and lipid components that exert their function at different stages of intestinal development and differentiation of intestinal cells. Obviously, genetic alterations linking these vital networks elicit malfunctions that are associated with severe disease phenotypes.

On September 4 and 5, 2016, a workshop on the molecular biology of the gut was held during the Congress of the German Society for Pediatric and Adolescent Medicine (DGKJ) in Munich and was generously sponsored by QOL Medical LLC, Vero Beach, FL, USA. This was a multidisciplinary workshop that combined molecular, cellular, immunological, and microbiological aspects of the gastrointestinal tract with clinical medicine and nutritional science. Experts viewed the gut as an organ from different angles and presented timely and comprehensive reviews on the impact of intestinal cellular and protein components and the corresponding basic mechanisms that maintain the functional capacities and the physiological hemostasis of the gut. The digestive function in the intestine, for example, is one of the most important and essential processes that are orchestrated by an organized and regulated network of hydrolases and transporters that are located in the microvillus membrane. Unraveling of the structural-functional relationships of these components as well as their biosynthetic and processing pathways has experienced a huge progress in the last few years, so that their malfunctioning and association with the onset of intestinal disorders and symptoms can be clarified. Particularly, the cell biology and pathobiochemistry of intestinal disaccharidases has occupied a major part of this workshop by virtue of the primordial function of the sucrase-isomaltase enzyme complex in carbohydrate and human starch digestion to glucose (α-glucogenesis) [1]. Sucrase-isomaltase is the major brush border enzyme with almost 5–7 % protein content of the entire brush border membrane and is the enzyme that is responsible for the digestion of almost all disaccharides that are transported to the intestinal lumen [2]. The expression of this protein during cellular differentiation as well as primary and secondary deficiencies has been among the topics discussed in this workshop [3]. Particularly, recent progress in identification of a large number of mutations associated with primary genetically determined sucrase-isomaltase deficiency (GSID or CSID) as well as various inheritance forms of this disorder has strongly suggested that this disorder is more common than initially thought. The implications of studying GSID is an important step towards developing therapies and envision approaches to botanical and technological alterations in food that will benefit all populations. Interestingly, the pathogenesis and the basic mechanisms that account for irritable bowel syndrome (IBS), one of the most common intestinal diseases, are until present obscure [4, 5]. Current concepts assume that aberrant or reduced functional capacities of intestinal disaccharidases, such as sucrase-isomaltase, are likely to be linked to the symptoms of IBS.

Not only is aberrant function of intestinal disaccharidases associated with chronic diarrhea, but also fructose malabsorption and insufficiencies in glucose transporters or NHE3 [6]. The workshop has also discussed the more global defects at the cellular levels such as in celiac disease, microvillus inclusion disease, or cystic fibrosis which also elicit chronic diarrhea [7–9]. Altogether, these disorders lead to substantial alterations of the intestinal microbiota and may alter and disturb the finely tuned intestinal hemostasis [10]. In fact, the gut mucosa is continuously exposed to bacterial and dietary antigens found in the lumen and microbial infections can stress and regulate the epithelial cell integrity and physiology.

The intestine brandishes a low-grade physiological inflammation, whereby the enterocytes and intraepithelial lymphocytes are essential in maintaining the gut hemostasis, since they constitute the first line of defense against pathogens and toxic molecules such as gliadin [8]. Under pathological conditions, such as the inflammatory bowel disease (IBD), the intestinal mucosa is infiltrated by inflammatory cells including neutrophils, macrophages, and lymphocytes. Here, the workshop covered the molecular genetics of IBD, the interaction between epithelial cells and lymphocytes, and addressed the question towards the regulation of enterocyte metabolism and proliferation by hypoxia-induced factors and stress [11, 12].

The presentations at the workshop were published in the peer-reviewed open-access journal *Molecular and Cellular Pediatrics* and are compiled in a printed version as a special issue. We are grateful to our colleagues for their outstanding contributions to the success of the workshop and for the excellent chapters in this book. We thank the German Society for Pediatric and Adolescent Medicine (DGKJ) for the financial support of this special issue.
